# Investigating the Role of Lactate-Related Genes in Radiotherapy Resistance of Lung Cancer by Integrated Bioinformatics and Experiment Validation

**DOI:** 10.7150/jca.113046

**Published:** 2025-07-24

**Authors:** Qianqian Li, Lili Shen, Yanguang Yang, Guomei Tai, Qiwei Zhu, Canyu Liu, Qin Ge, Qiong Yi

**Affiliations:** 1 Department of Radiation Oncology, Nantong Tumor Hospital, Affiliated Tumor Hospital of Nantong University, Nantong 226361, China.; 2 Department of Oncology, Haimen People's Hospital, Nantong 226100, China.; 3 Department of Radiation Oncology, The Fourth Affiliated Hospital of Soochow University, Suzhou, Dushu Lake Hospital, Medical Center of Soochow University, Suzhou 215000, China.

**Keywords:** lung cancer, radiotherapy resistance, lactate regulator, risk score, FADS2

## Abstract

Radiotherapy is a standard treatment for advanced lung cancer, but resistance remains a significant cause of treatment failure. This study aimed to investigate lactate-associated genes to identify patients likely to benefit from radiotherapy. RNA-seq data from 99 patients with lung cancer who underwent radiotherapy were analyzed to identify differentially expressed genes (DEGs) between resistant and sensitive cases. Bioinformatics tools were used to assess the prognostic relevance of lactate-related genes, and a risk score model was develpoed based on these genes. Dysregulation of these genes in patients with lung cancer undergoing radiotherapy was validated through *in vitro* experiments. Molecular docking was used to explore potential radiosensitizers. The analysis identified 1482 DEGs, with enrichment analysis highlighting lactate metabolism pathways. A risk score model was constructed using the lactate-related genes ADAMTS3, FADS2, and RTBDN to classify patients into high- and low-risk subgroups. Functional enrichment analysis revealed the model's impact on DNA repair and tumor immunity. A nomogram was developed for clinical implementation. Wet lab experiments further confirmed these findings. In conclusion, a novel risk score model based on lactate-related genes was developed to predict radiotherapy outcomes in lung cancer. FADS2 was identified as a potential biomarker for predicting resistance to radiotherapy. This study is the first to examine the predictive value of lactate-related genes for radiotherapy efficacy in lung cancer, offering valuable insights for personalized treatment strategies to improve therapeutic outcomes.

## Introduction

Lung cancer is the most prevalent and fatal malignancy worldwide, with a five-year overall survival (OS) rate of only 19%^1^, ^2^. Due to its subtle symptoms, most patients are diagnosed at advanced stages, resulting in poor prognoses^3^. Consequently, for advanced lung cancer, the primary treatment options include radiotherapy in combination with targeted therapy, chemotherapy, and immunotherapy, all of which have significantly improved survival rates^4^, ^5^. However, radiotherapy resistance remains the predominant cause of treatment failure. Thus, understanding the molecular mechanisms underlying radiotherapy resistance is crucial for developing more effective strategies to improve patient outcomes.

Radiotherapy kills tumor cells primarily through the generation of reactive oxygen species (ROS) and DNA breakage^6^. However, tumor heterogeneity leads to the survival of residual tumor cells, which can repair DNA, activate immune responses, promote cancer stem cells, and suppress various forms of cell death, all of which contribute to radiotherapy resistance^6^-^8^. Notably, all these processes require energy, which is often provided through altered metabolism. The Warburg effect, a hallmark of tumor metabolism, promotes rapid energy production *via* aerobic glycolysis, resulting in a lactic acid microenvironment^9^. Glycolysis has been linked to tumor metastasis and resistance to both chemotherapy and radiotherapy^10^-^13^. Lactate plays a pivotal role in delivering oxidative and gluconeogenic substrates and transducing cellular signaling in these processes^14^, ^15^. In lung cancer, lactate-related genes have been shown to influence prognosis and immune response^16^-^18^. However, studies investigating the regulation of lactate-related genes in the context of radiotherapy resistance in lung cancer remain scarce.

This study identified three prognostic lactate-related differentially expressed genes (LRDs) associated with radiotherapy resistance in patients with lung cancer from TCGA data. A risk score model based on these LRDs was developed and validated for clinical application, and the related mechanisms were further explored through bioinformatics analysis and *in vitro* experiments. Our findings suggest that lactate regulators could serve as biomarkers for predicting radiotherapy resistance in patients with lung cancer, providing clinicians with valuable tools to tailor personalized treatment strategies and improve therapeutic outcomes.

## Materials and Methods

### Data collection and sample preprocessing for radiotherapy patients with lung cancer

RNA sequencing, survival data, and clinical phenotypes were obtained from The Cancer Genome Atlas (TCGA) database. A total of 522 primary lung adenocarcinoma patients were obtained and underwent screening. The inclusion criteria were listed as following conditions: (a) patients with postoperative radiotherapy; (b) patients who have an evaluation of the efficacy of radiotherapy or information about their progress after radiotherapy; (c) patients who have a clear state of alive or death and a definite survival time. The exclusion criteria were listed as following conditions: (a) the patients whose number in the expression profile didn't match with the patient number corresponding to the clinical information (n=9); (b) patients with new primary tumor(n=12); (c) patients without postoperative radiotherapy or missing evaluation of the efficacy of radiotherapy or information about their progress after radiotherapy (n=389); (d) patients missing a clear state of alive or death, or a definite survival time (n=13). Finally, a cohort of 99 patients was extracted. Among them, 35 patients exhibited a complete response or partial response to radiotherapy, were categorized as the radiosensitive group. While 64 patients, exhibited progressive disease or stable disease or recurrence after radiotherapy, were included in the radioresistant group. Single cell sequencing analysis was performed on the lung cancer tissues in GSE179373.

### Identification of differentially expressed genes (DEGs) and functional enrichment analysis

The threshold value for screening DEGs was set to |FC| > 1.2 and P < 0.05^19^. Gene Ontology (GO) and Kyoto Encyclopedia of Genes and Genomes (KEGG) pathways of DEGs were analyzed to determine their potential functions and pathways. GO analysis, including biological processes, cellular components, and molecular functions, helped to understand the biological functions as well as positioning of DEGs. KEGG pathway analysis clarified the signaling pathways that DEGs may focus on. P < 0.05 was considered to be statistically significant. Gene set enrichment analysis (GSEA), and gene set variation analysis (GSVA) were conducted among DEGs using the R packages.

### Construction of lactate-related risk score model

After lactate-related genes were downloaded from genecards database and were intersected with DEGs, LRDs were analyzed by Univariate COX regression analysis. Then, least absolute shrinkage and selection operator (LASSO) algorithm was performed on the significant LRDs in radiotherapy patients with lung cancer in TCGA cohort. Finally, the lactate related risk score model was constructed by the stepwise Cox regression algorithm. Radiotherapy patients with risk-score above the median were categorized as the high-risk subgroup, and the rest were included in the low-risk subgroup. For model, the time dependent receiver operating characteristic (ROC) was calculated. Survival analysis was used “survival”, “survminer” and “timeROC” packages, and the nomogram was used “rms” package.

### Immune infiltration analysis

Cibersort algorithm analyzed the proportion of 22 immune cells in high-risk and low-risk subgroups. ssGSEA, xcell, ESTIMATE detected the different distribution of the immunity cells between the high-risk subgroup and low-risk subgroup using the SangerBox 3.0 platform.

### Methylation and mutation analysis

The assessment of methylation status in ADAMTS3, FADS2, RTBDN between tumor and their corresponding para-cancerous tissues was conducted by the UALCAN database. The maftools package in R software was taken to download and extract mutation data, and the somatic mutation data of patients in high-risk subgroup and low-risk subgroup were then visually analyzed.

### Immunohistochemical (IHC) analysis

The study was approved by the Research Ethics Board of the Tumor Hospital Affiliated with Nantong University (No.2023-053). Tissues slice was incubated with a primary antibody against a 1:100 dilution of FADS2 (Rabbit-anti-human, Proteintech) overnight at 4° C after the dewaxing and blocking of endogenous peroxides of the tissues. Visualisation of the antibody complex was achieved through a diaminobenzidine reaction. Tissue was counterstained by Meyer's haematoxylin. Based on the intensity of staining in the tumor from three hot spots of each tissue, IHC staining was analyzed by pathologists. Image J was used to score the intensity of staining.

### Quantitative real-time PCR (qPCR)

RNA was isolated from H1299 radioresistant cell and radiosensitive cell A549^20^, ^21^ by Trizol. Reverse transcription and quantitative PCR were carried out by using a two-step Prime Script TM RT reagent kit (TAKARA). Primers for the genes were synthesized and obtained from Thermo Fisher Scientific. The primer sequences are presented in **Supplementary Table 1.**

### Cell culture and clonogenicity assay

The human lung cancer cell lines H1299, A549 were purchased from ATCC. Cell lines were maintained under a humidified atmosphere of 5% CO2 in air at 37℃ in RPMI 1640 medium supplemented with 10% FBS (Gibco BRL, USA) and 1% penicillin-streptomycin. H1299 or H1299 with lentivirus-mediated FADS2 overexpression were plated in 6-well plates prior to ionization radiation exposure (4 Gy, 8 Gy), and maintained in culture for 14 days. Afterward, when colonies were formed (defined as a bulk of at least 50 cells), cells were stained by crystal violet reagents.

### Weighted gene co-expression network analysis (WGCNA)

The co-expression network of radiotherapy patients with lung cancer in TCGA was generated using WGCNA package. A suitable soft threshold β is calculated based on the criteria for scale-free networks. In the following step, the weighted adjacency matrix was converted into a topological overlap matrix (TOM), and the corresponding dissimilarity (1-TOM) was calculated. Module identification was conducted using the dynamic tree cutting approach. The modules most relevant to the clinical phenotype were selected for subsequent analysis.

### Molecular docking

In order to estimate the likelihood of drug interactions, the DEGs was identified from the high- and low-risk subgroups. L1000FWD database was used to detect the signaling pathways affected by small molecule drugs. Drug-gene interactions were performed on the Network Analyst Database. Furthermore, Home for researches platform was used for visualizing the molecular docking.

### Statistical analysis

SPSS 20.0 software was used for statistical analysis, and Graphpad Prism 9 software for drawing the survival curve and statistical chart of the difference between the two groups. The difference was tested by independent sample t-test. Cox proportional hazards regression model was taken for univariate and multivariate analysis, while Kaplan Meier method and Log-rank test for overall survival analysis. P < 0.05 was considered significant.

## Results

### Identification of the key biological processes and signaling pathways to radiotherapy resistance in lung cancer

**Figure 1:**
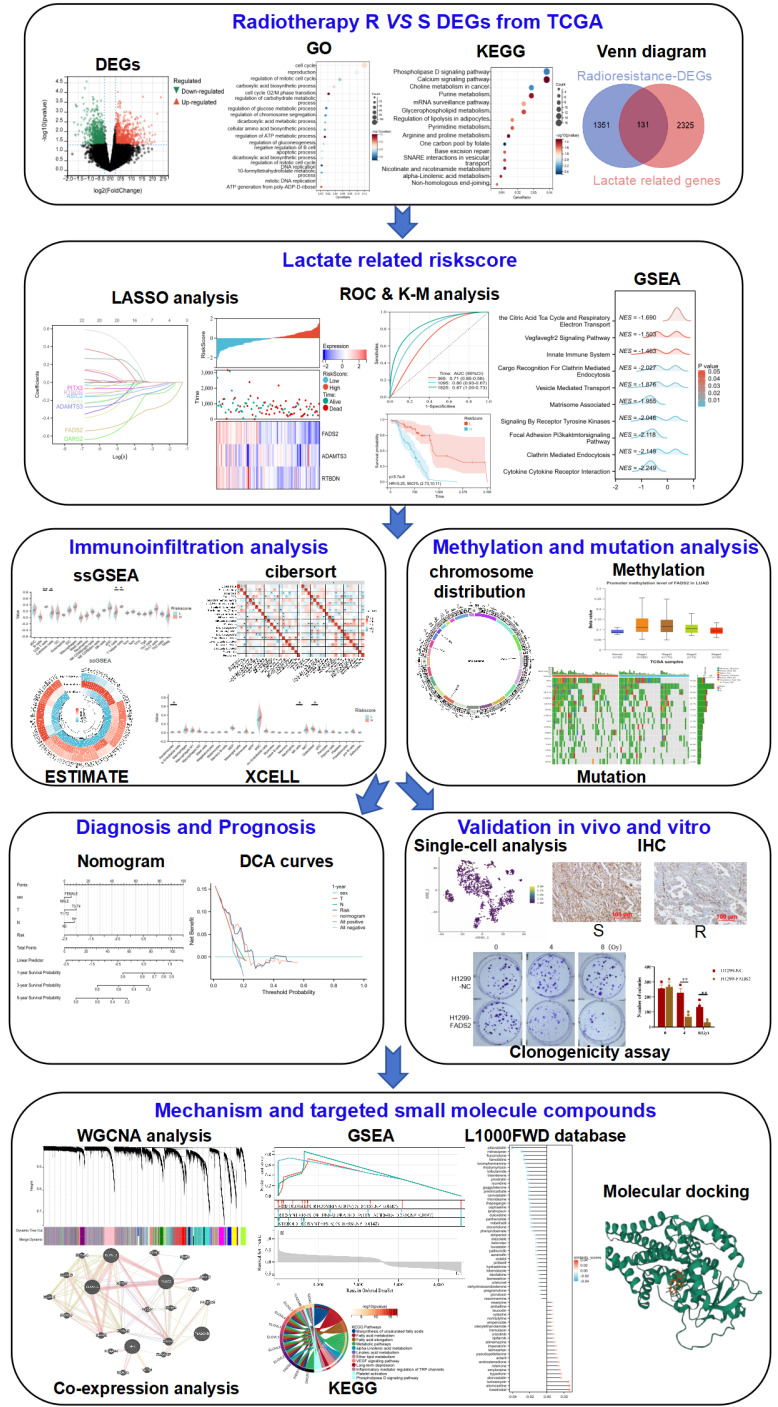
** The flow chart of the research.** R: resistance, S: sensitive, DEGs: differentially expressed genes.

**Figure 2:**
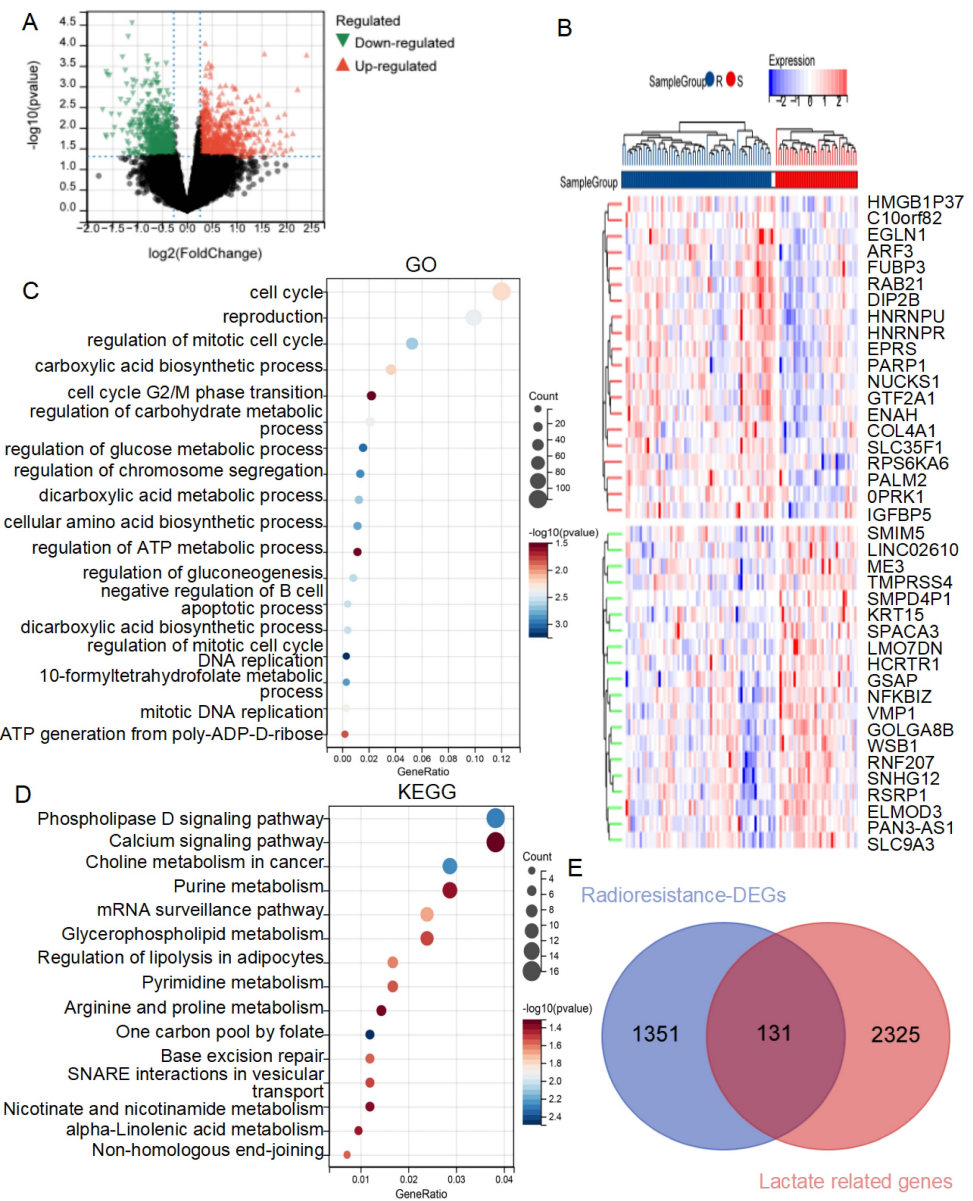
** GO and KEGG analysis of DEGs from radioresistant patients comparing with sensitive ones with lung cancer in TCGA. (A)** Volcano plot of the 1,482 DEGs. The red triangles represent the significantly up-regulated genes, and the blue triangles showed the significantly down-regulated genes.** (B)** Heat map showed the TOP20 up-regulated and down-regulated genes respectively.** (C)** GO enrichment analysis of the DEGs.** (D)** KEGG enrichment analysis of the DEGs.** (E)** The venn diagram of radiotherapy resistance DEGs and lactate-related genes.

The workflow of this study is illustrated in **Figure 1**. To investigate the mechanisms of radiotherapy resistance in lung cancer, DEGs between radiotherapy-resistant and sensitive patients from the TCGA cohort were analyzed using limma. A total of 1482 DEGs (633 downregulated and 849 upregulated) as showed in volcano plot **(Figure 2A)**, with the top 20 upregulated and downregulated DEGs displayed in the heatmap **(Figure 2B)**. Further analysis of these DEGs using GO and KEGG enrichment revealed significant biological processes. GO analysis identified three primary categories: DNA damage repair (including the cell cycle, G2/M phase transition, and DNA replication)^22^, ^23^, lactate metabolism (including regulation of carbohydrate metabolism, gluconeogenesis, and ATP generation from poly-ADP-D-ribose)^24^, ^25^, and immune cell regulation (specifically the negative regulation of B cell apoptosis)** (Figure 2C)**. KEGG analysis highlighted pathways associated with the Warburg effect, including phospholipase D signaling, glycerophospholipid metabolism, one-carbon pool by folate, and alpha-linolenic acid metabolism, as well as excision repair pathways^26^-^28^** (Figure 2D)**. These findings highlight the critical roles of lactate metabolism, DNA repair, and immune response in radiotherapy resistance.

### Construction of the lactate-related risk score model for radiotherapy patients with lung cancer

**Figure 3:**
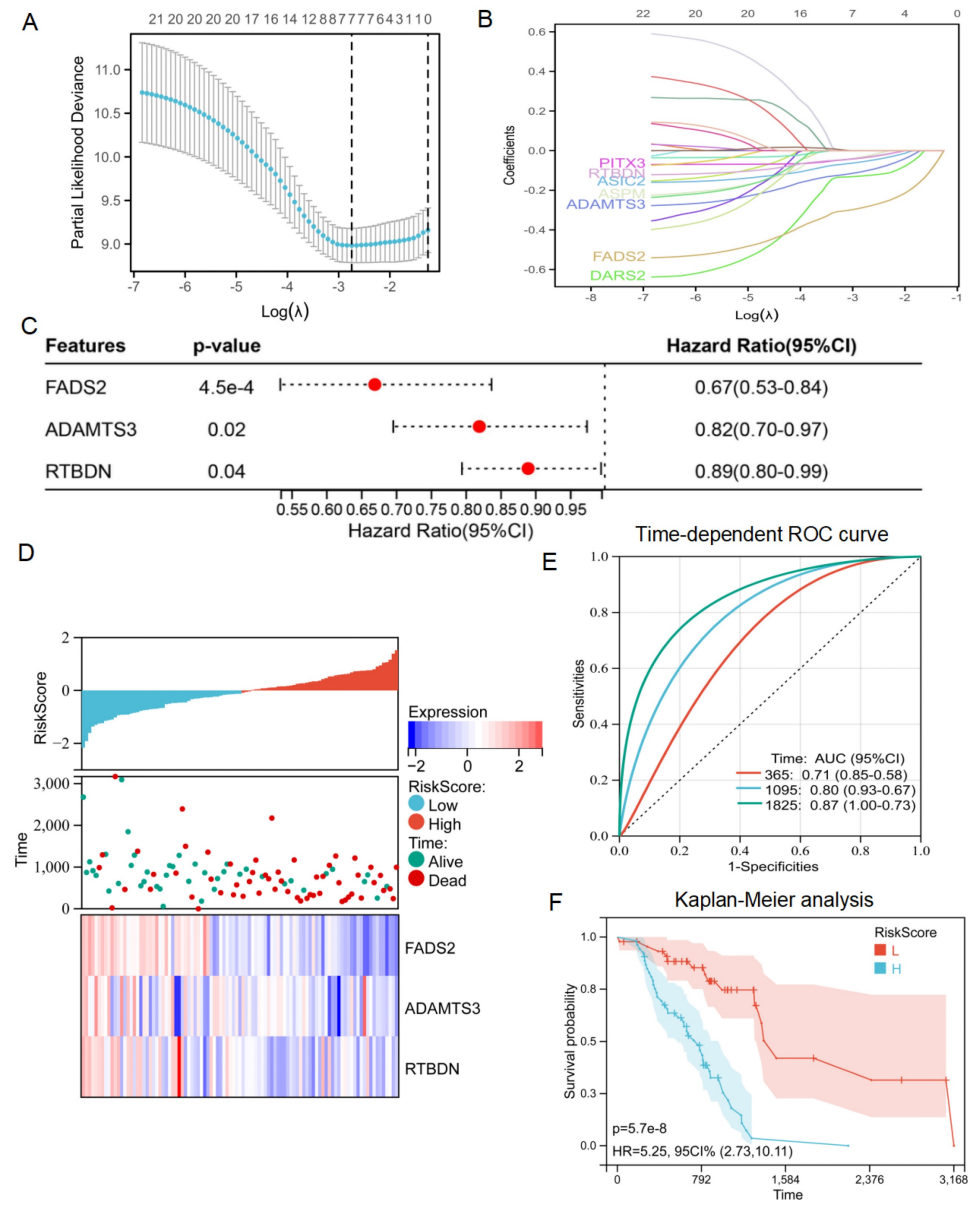
** Identification of lactate related signature via LASSO-stepwise algorithms. (A, B)** LASSO analysis with minimal lambda value. **(C)** Three genes were screen out by stepwise Cox algorithm. **(D)** Lactate-related risk score model illustrating the distribution of survival status and the expression of the three candidate genes.** (E)** The time-dependent ROC curve for lactate-related risk score. **(F)** Kaplan-Meier analysis demonstrated the prognostic significance of the risk model in TCGA.

Based on these results, an intersection analysis of lactate-related genes and DEGs, identified 131 LRDs **(Figure 2E)**. Subsequently, univariate Cox regression analysis was employed to construct a risk stratification system, with 23 LRDs found to be prognostically relevant for radiotherapy patients in the TCGA cohort **(Table 1)**. To prevent overfitting of the prognostic signature, LASSO regression was applied to these 23 prognosis-related LRDs, resulting in the selection of seven LRDs based on the optimal λ value **(Figures 3A, 3B)**. Stepwise regression further refined the model, extracting three key genes **(Figure 3C)**. The risk score model was formulated using the following algorithm: risk score = (-0.257131817660543) × ADAMTS3 + (-0.500985047) × FADS2 + (-0.136319284) × RTBDN. The radiotherapy patients in the TCGA cohort were divided into high- and low-risk subgroups based on the median risk score for subsequent analysis. Comparisons of risk score distributions and survival statuses between these subgroups were performed **(Figure 3D)**. The specificity and sensitivity of the signature were assessed by calculating the area under the curve (AUC) values for 1-, 3-, and 5-year survival, yielding AUCs of 0.71, 0.80, and 0.87, respectively, in the TCGA cohort **(Figure 3E)**. Survival analysis revealed that patients in the high-risk subgroup had significantly poorer prognosis **(**P = 5.7e-8; **Figure 3F)**.

### Functional enrichment analysis of different risk score subgroups

**Figure 4:**
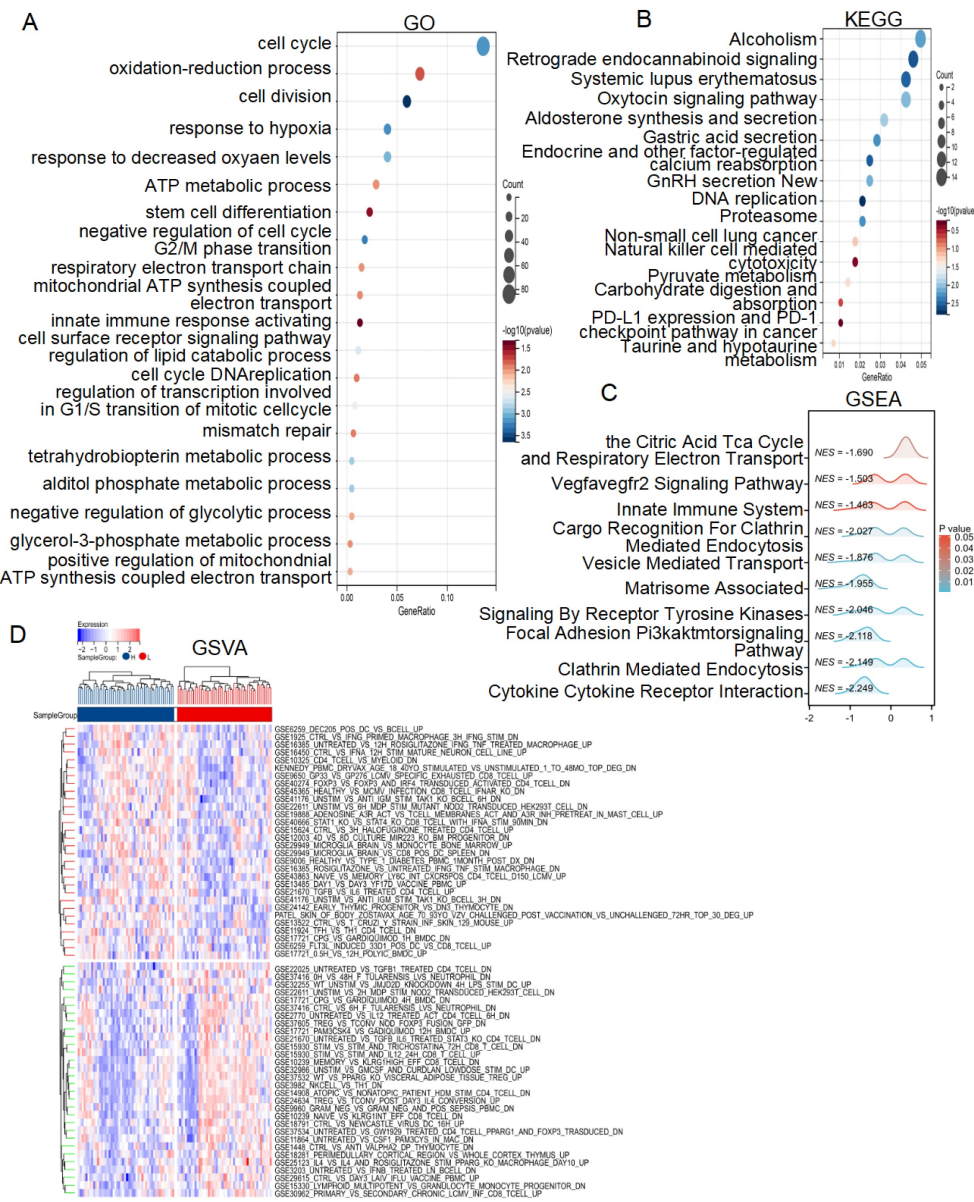
** Enrichment analysis of the lactate-related risk score.** GO** (A)**, KEGG **(B)**, GSEA** (C)** and GSVA** (D)** analysis of the DEGs and potential signaling pathways in different lactate-related risk score subgroups.

To investigate the molecular mechanisms underlying the different risk score subgroups, we performed a DEGs analysis of patients from the TCGA cohort and identified 1067 genes (|FC| > 1.2, P< 0.05). GO analysis revealed that the lactate-related risk score primarily impacted processes such as the cell cycle, DNA replication, oxidation-reduction processes, response to hypoxia, ATP metabolic process, mitochondrial ATP synthesis coupled electron transport, innate immune response activating cell surface receptor signaling pathway, regulation of lipid catabolic process, tetrahydrobiopterin metabolic process, alditol phosphate metabolic process, negative regulation of glycolytic process, glycerol-3-phosphate metabolic process **(Figure 4A)**. KEGG analysis highlighted pathways involved in DNA replication, non-small cell lung cancer, natural killer cell mediated cytotoxicity, pyruvate metabolism, carbohydrate digestion and absorption-PD-L1 expression and PD-1 check point pathway in cancer **(Figure 4B)**. Additionally, the GSEA algorithm explored the citric acid TCA cycle and respiratory electron transport, VEGFA-VEGFR2 signaling, innate immune system pathways, cargo recognition for clathrin mediated endocytosis, vesicle mediated transport, matrisome associated signaling by receptor tyrosine kinases were different between high- and low-risk score subgroups **(Figure 4C)**. GSVA algorithm further revealed differences in multiple immune regulatory pathways between the subgroups **(Figure 4D)**. These results suggest that lactate-related risk scores modulate radiotherapy outcomes through energy metabolism, immune infiltration, and DNA repair mechanisms.

### Immune infiltration analysis for lactate-related risk score

**Figure 5:**
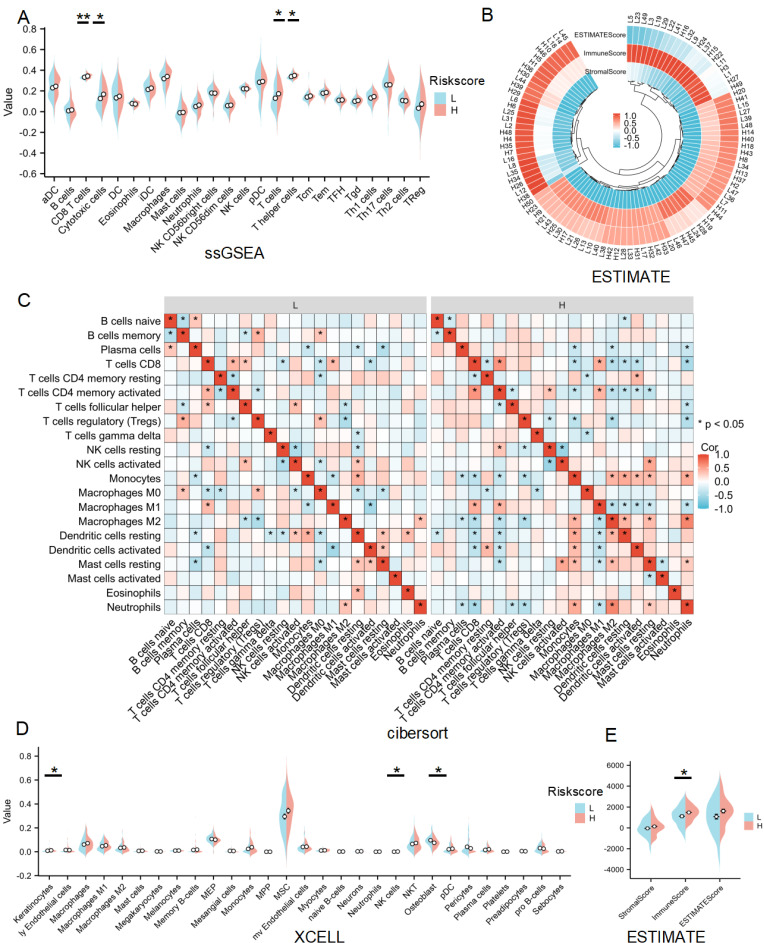
** Immune infiltration analysis of lactate-related risk score. (A)** ssGSEA analysis detected immune cell expression between the high- and low-risk score subgroups.** (B, E)** The landscape and comparing of the immunoscore in high- and low-risk score subgroups by ESTIMATE algorithms.** (C)** CIBERSORT showed the different correlations between 22 immune cells in the high- and low-risk score subgroups, respectively. **(D)** Xcell algorithms detected immune cell expression between the high- and low-risk score subgroups. L:low-risk score subgroup, H: high-risk score subgroup, *P<0.05, **P<0.01.

Given that GO, KEGG, GSEA and GSVA analyses all indicated a significant role of LRDs in immune infiltration, immune cell infiltration was assessed across 24 cell types using ssGSEA **(Figure 5A)**. The results showed that CD8+ T cells, cytotoxic cells, T cells, and T helper cells were significantly more abundant in the high-risk score subgroup compared to the low-risk subgroup. ESTIMATE analysis showed the score of each sample **(Figures 5B)** and found immune score was higher in the high-risk subgroup **(Figures 5C)**. CIBERSORT algorithms confirmed that CD4+ memory-activated T cells and M1 macrophages were inhibited, whereas M2 macrophages were significantly activated in the high-risk subgroup **(Figures 5D)**. XCELL algorithm explored differences in Keratinocytes, NK cells and Osteoblast between the two subgroups **(Figure 5D)**. Collectively, these results highlight the distinct patterns of immune cell infiltration between the lactate-related risk score subgroups, suggesting that immune modulation may influence the effectiveness of radiotherapy in patients with lung cancer.

### Methylation and mutation analysis for lactate-related risk score

**Figure 6:**
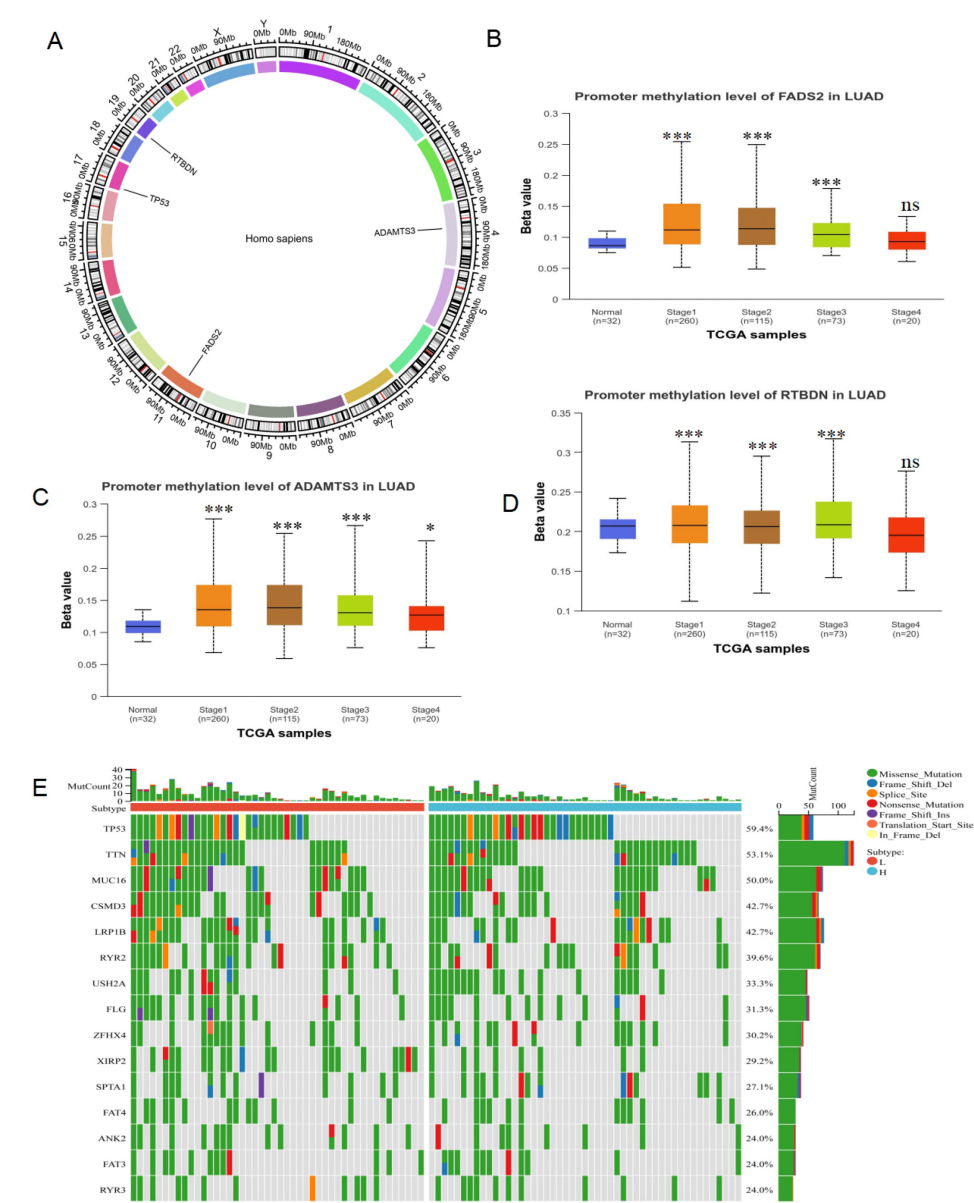
** Methylation and mutation analysis of LRDs. (A)** The chromosomal distribution of LRDs by circos plots. Methylation analysis of FADS2 **(B)**, ADAMTS3** (C)** and RTBDN** (D)** in different stage of lung cancer and normal tissues was explored in the UALCAN database.** (E)** The top 15 mutation genes in high and low lactate-related risk score subgroups, respectively. ***:P < 0.001.

To explore the differences between lactate-related risk score subgroups, the chromosomal distribution of the three genes used to construct the lactate-related risk score was visualized through circos plots **(Figure 6A)**. Given the role of aberrant DNA methylation in cancer-related dysregulation, the UALCAN database was utilized to investigate the relationship between the expression patterns of ADAMTS3, FADS2, and RTBDN and methylation levels in both normal and tumor tissues. The increased methylation of ADAMTS3, FADS2 and RTBDN was found in lung cancer tissues, potentially contributing to their downregulation **(Figure 6B-6D)**. The gene mutation landscape revealed significant mutations in TP53, TTN, MUC16, USH2A, and ZFHX4 in the high-risk score subgroup, and in CSMD3, LRP1B, XIRP2, and RYR3 in the low-risk score subgroup **(Figure 6E)**.

### Clinical applications for lactate-related risk score

**Figure 7:**
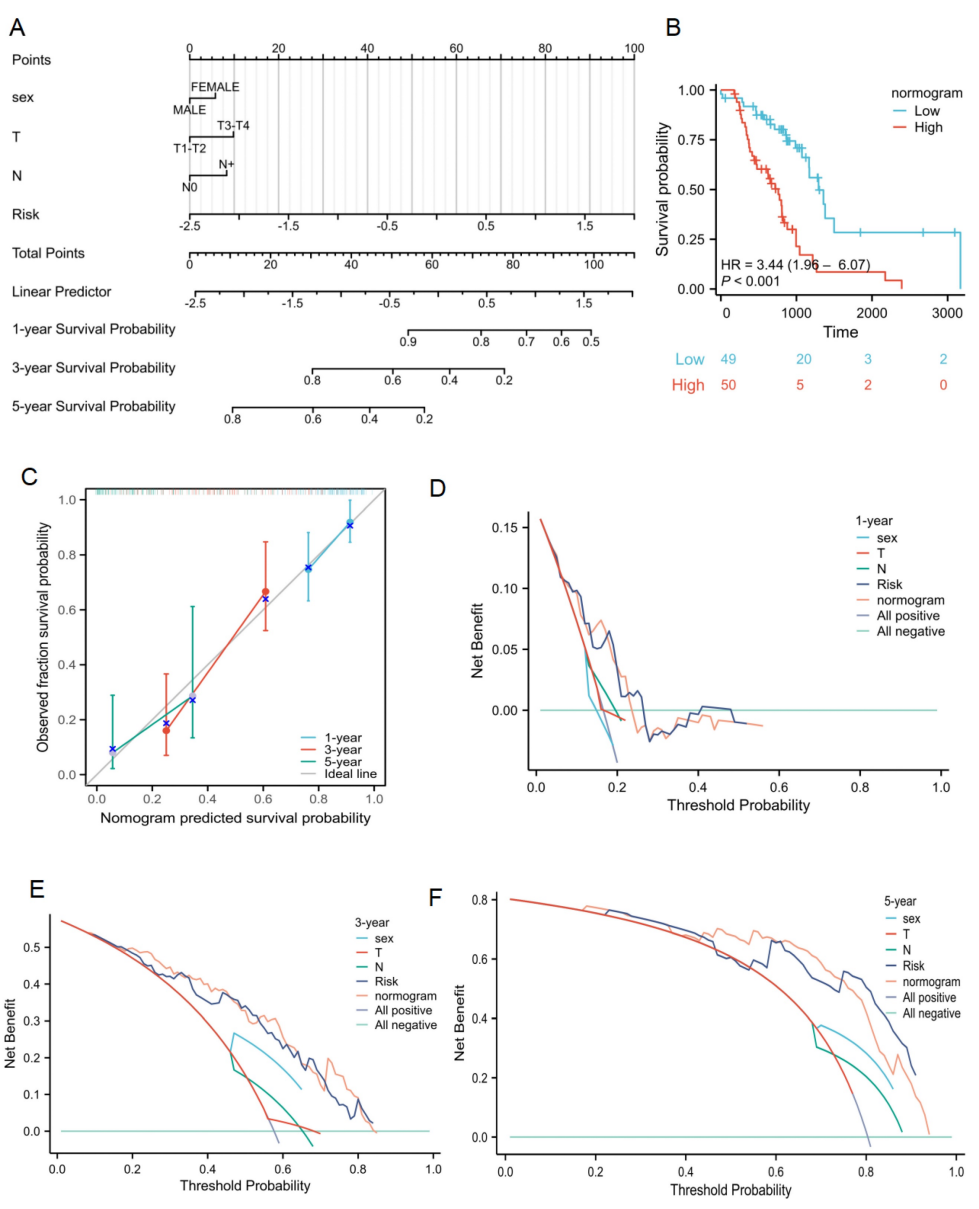
** Clinical application of lactate-related risk score. (A)** The nomogram of the risk score and clinical parameters for radiotherapy patients with lung cancer in TCGA cohort. **(B)** Kaplan-Meier plot analysis based on nomogram. **(C)** The calibration curves displayed the accuracy of the nomogram in the 1-, 3-, and 5-years. **(D-F)** DCA curves to assess the ability of sex, T stage, N stage, risk score, and their combination to predict 1-, 3-, 5-years overall survival of radiotherapy patients with lung cancer in TCGA cohort.

The relationship between the lactate-related risk score and clinical parameters was also assessed. The results showed significant correlations between the risk score and sex, radiotherapy response, T status, and N status (P < 0.001, **Supplementary Table 2**). To capture the complexity of the risk signature, a nomogram was developed incorporating both clinical information and the risk scores of radiotherapy patients with lung cancer from the TCGA cohort** (Figure 7A)**. Patients with high risk scores had poorer survival compared to those with low scores **(Figure 7B)**. Calibration plots for OS outcomes demonstrated strong concordance between predicted and observed OS at 1-, 3-, and 5-year intervals. The C-index for prediction was 0.701 (0.661-0.741), indicating a relatively good predictive performance **(Figure 7C)**. Additionally, the nomogram model was evaluated using decision curve analysis (DCA) for radiotherapy patients with lung cancer in the TCGA cohort **(Figure 7D-7F)**. Collectively, the lactate-related risk score emerged as an independent and robust prognostic indicator, offering enhanced predictive value when combined with clinical features for radiotherapy patients with lung cancer.

### Expression and prognosis of LRDs in radiotherapy patients with lung cancer

**Figure 8:**
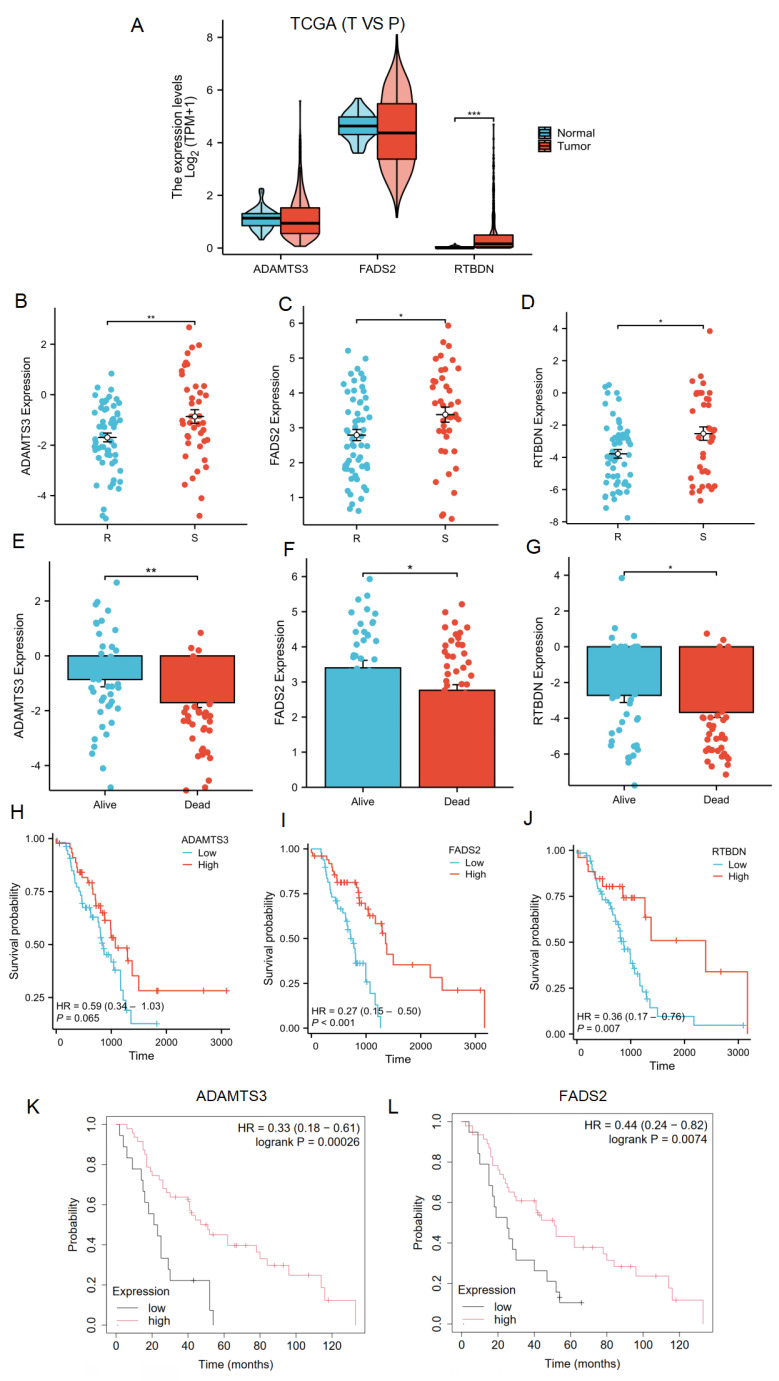
** Expression and prognostic significance of LRDs. (A)** Expression of LRDs in lung cancer patients of TCGA. **(B-G)** Expression of LRDs in radiotherapy patients with lung cancer of TCGA cohort. **(H-J)** Correlation between LRDs and OS in radiotherapy patients with lung cancer of TCGA cohort.** (K-L)** Correlation between LRDs and OS in radiotherapy patients with lung cancer from KM-plot database.

The lactate-related risk score, constructed using ADAMTS3, FADS2, and RTBDN, was further explored to assess their expression and prognostic value for radiotherapy patients with lung cancer across multiple databases. ADAMTS3 and FADS2 exhibited downregulation in tumor tissues compared to normal lung tissues, whereas RTBDN displayed an opposite trend **(Figure 8A)**. Notably, the expression of these three genes was significantly downregulated in the radiotherapy-resistant group from the TCGA cohort **(Figure 8B-8D)**, as well as in the deceased subgroup from the same cohort **(Figure 8E-8G)**. Survival analysis revealed that radiotherapy patients with low FADS2 expression had a poorer prognosis compared to those with high expression of FADS2 in the TCGA cohort (P < 0.001). A similar trend was observed for ADAMTS3 (P = 0.065) and RTBDN (P = 0.007) **(Figure 8H-8J)**. Moreover, validation in the KM-Plot database confirmed that radiotherapy patients with low expression of FADS2 and ADAMTS3 had better prognoses, whereas RTBDN did not show significant prognostic relevance **(Figure 8K-8L)**. Based on these findings, FADS2 was prioritized for further validation.

### Validation of the LRDs in lung cancer tissues and radioresistant cells

**Figure 9:**
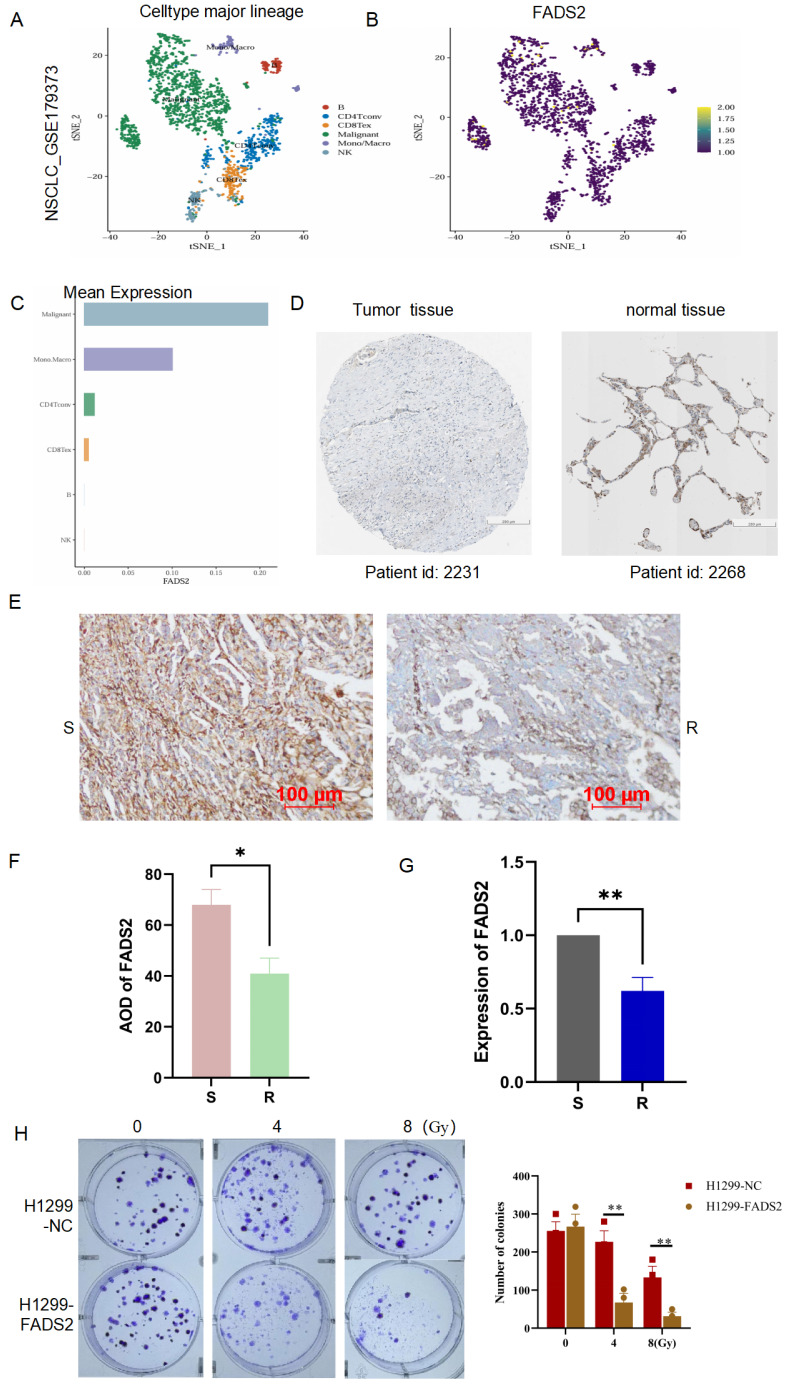
** Validation the expression and role of FADS2 in radiotherapy resistance. (A, B, C)** Single-cell sequencing analysis for the location and expression of FADS2 in lung cancer tissue from GSE179373.** (D)** IHC staining of FADS2 in lung cancer and para-carcinoma tissue in HPA database.** (E&F)** IHC analysis of FADS2 in radioresistant (n=5) and radiosensitive (n=5) tissues. **(G)** qPCR analysis of FADS2 in radioresistant cell (H1299) and radiosensitive cell (A549). **(H)** Clonogenicity assay explored the role of FADS2 in radiotherapy sensitive. H1299-FADS2: over-expression of FADS2 in H1299. *:P < 0.05, **:P < 0.01.

To explore the expression and localization of FADS2 in lung cancer tissues, we analyzed single-cell RNA sequencing data from lung cancer (GSE179373) were analyzed, revealing that FADS2 was predominantly expressed in malignant cells **(Figure 9A-9C)**. In the Human Protein Atlas database, the expression of FADS2 was lower in lung cancer tissues compared to normal lung tissues **(Figure 9D)**. To further validate these findings, 10 lung cancer tissue samples (5 radioresistant and 5 radiosensitive) were collected, and IHC analysis showed significantly lower FADS2 expression in radioresistant tissues compared to radiosensitive tissues **(Figure 9E, 9F)**. At the cellular level, qPCR confirmed a significant decrease in FADS2 expression in radioresistant cells **(Figure 9G)**. Furthermore, colony formation assays demonstrated that upregulation of FADS2 significantly increased the sensitivity of radioresistant cells to 4Gy and 8Gy irradiation **(Figure 9H)**. Collectively, these results suggest that FADS2 is associated with radiotherapy resistance in lung cancer.

### Exploring the potential mechanism of LRDs regulating radiotherapy resistance in lung cancer

**Figure 10:**
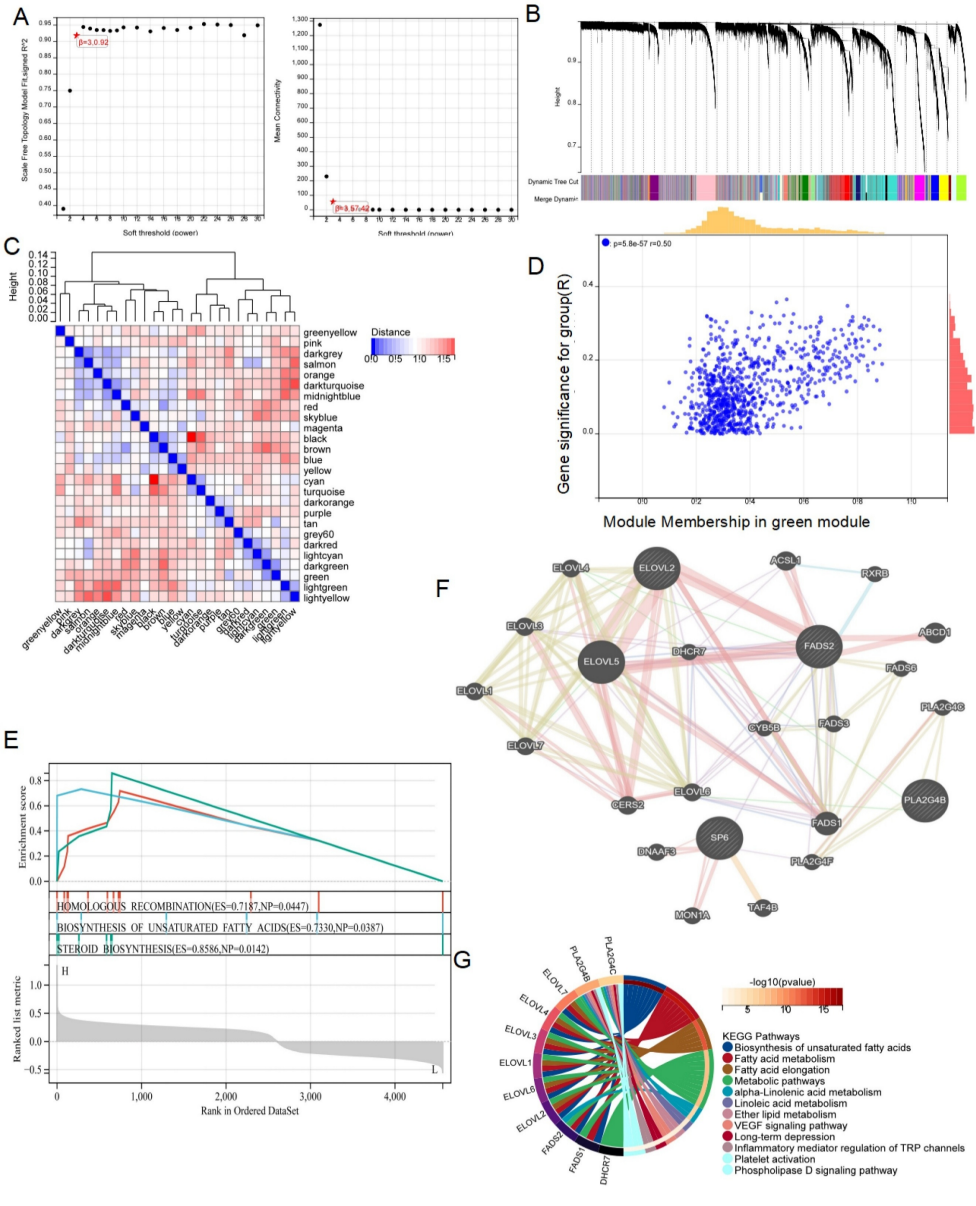
** Identifying the role of FADS2 via WGCNA analysis. (A)** The correlation between soft threshold and scale free topology model as well as mean connectivity. **(B)** Highly interconnected groups of genes were clustered and modules are represented by distinct colours in the horizontal bar. **(C)** The correlations between different modules were calculated. **(D)** The high correlation between GS and MM in the green module in radiotherapy resistance.** (E)** Genes in the green module were analyzed by GSEA. **(F)** Co-expression analysis for FADS2 with genes in the green module.** (G)** KEGG enrichment analysis for the key genes co-expressed with FADS2.

To further elucidate the mechanism by which FADS2 regulates radiotherapy resistance in lung cancer, WGCNA was conducted based on the DEGs from the TCGA cohort, stratified by the median FADS2 expression. A soft threshold of β = 3 was applied to construct a scale-free network** (Figure 10A)**. The DEGs were grouped into 26 co-expression modules, represented in various colors **(Figure 10B, 10C)**. The green module exhibited the strongest correlation with radiotherapy resistance, with a module-trait relationship R value of 0.50 (P = 5.8e-17) **(Figure 10D)**. Then, 875 DEGs from the green module underwent GSEA revealing enrichment in pathways such as homologous recombination, biosynthesis of unsaturated fatty acids, and steroid biosynthesis in the FADS2-high expression group **(Figure 10E)**. Additionally, the GeneMANIA database identified co-expressed genes within the green module associated with FADS2 **(Figure 10F)**, which were enriched in biosynthesis of unsaturated fatty acids fatty acid metabolism, fatty acid elongation, metabolic pathways, linoleic acid metabolism, ether lipid metabolism, VEGF signaling pathway, inflammatory mediator regulation of TRP channels, platelet activation, and phospholipase D signaling pathway **(Figure 10G)**.

### Molecular docking of small molecule compounds for improving radiotherapy effect

**Figure 11:**
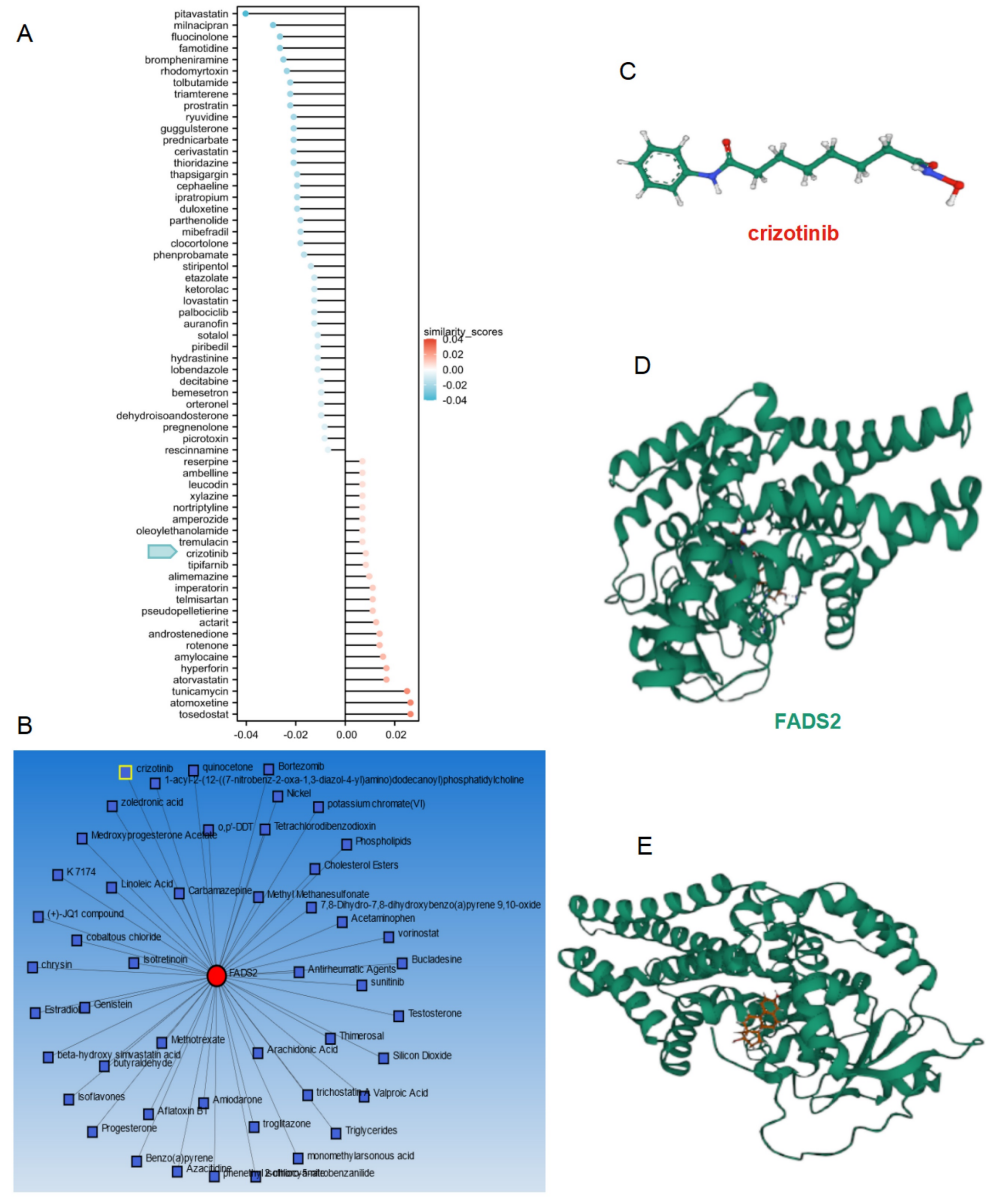
** Drug analysis and molecular docking for inhibiting radiotherapy resistance in lung cancer. (A)** The potential drugs for lung cancer treatment. **(B)** Network analyst database predicting the potential drugs reacting with FADS2. **(C)** The 2D structure of crizotinib. **(D)** The protein structure of FADS2. (E) Molecular docking for crizotinib and FADS2.

To improve the prognosis of patients in the high-risk score subgroup, the L1000FWD database was utilized to identify small-molecule compounds with potential therapeutic effects **(Figure 11A)**. The NetworkAnalyst database was employed to explore compounds targeting FADS2 **(Figure 11B)**. Crizotinib emerged as a common drug in both databases, exhibiting inhibition of FADS2 expression. The structure of crizotinib and its interaction with FADS2 were visualized **(Figure 11C-11E)**. Molecular docking analysis confirmed the direct interaction between FADS2 and crizotinib. The hydrophobic pockets of both targets were successfully occupied, with a low binding energy of -7.896 kcal/mol, indicating a highly stable binding. These results suggest that crizotinib may serve as a potential radiosensitizer for lung cancer.

## Discussion

Lung cancer, a leading cause of cancer-related mortality globally, presents a significant challenge in clinical management. Radiotherapy plays a critical role in managing advanced-stage lung cancer, offering symptom relief and controlling disease progression^29^. However, the development of radiotherapy resistance remains a substantial barrier, contributing to rapid disease progression. Understanding the mechanisms underlying this resistance, particularly the role of lactate metabolism, is essential for developing strategies to overcome this challenge.

This study identified DNA repair and lactate metabolism-associated biological processes and signaling pathways as key factors in radiotherapy resistance in lung cancer. On one hand, DNA repair is a well-established contributor to radiotherapy resistance^30^, supporting the validity of our findings. On the other hand, this result underscores the importance of lactate-related genes in modulating the radiotherapy response.

Building on these insights, LRDs were identified, and a lactate-related risk score model was constructed using LASSO regression and stepwise Cox regression. Time-dependent ROC and survival analyses demonstrated the strong predictive and diagnostic value of the risk score in patients with lung cancer undergoing radiotherapy. To explore the underlying mechanisms, GO, KEGG, GSEA, and GSVA analyses confirmed a significant enrichment of biological processes related to energy metabolism, including response to hypoxia, ATP metabolism, respiratory electron transport, and lipid catabolic regulation. Under hypoxic conditions, the hypoxia-inducible factor (HIF) induces LDHA expression, driving anaerobic metabolism by converting pyruvate to lactate to generate ATP, which enhances cell proliferation and defines radiotherapy resistance in prostate cancer^31^. The Warburg effect, which persists even under normoxic conditions, promotes DNA repair and enhances cell survival, ultimately contributing to resistance in various cancers, including head and neck cancer^32^, esophageal squamous cell carcinoma^33^, lung cancer^34^. These findings suggest that lactate-related genes may regulate radiotherapy resistance in lung cancer by promoting the Warburg effect, thus providing energy necessary for DNA repair.

Additionally, this study revealed differences in immune cell infiltration between lactate-related risk score subgroups. CD4 memory T cells and M1 macrophages were inhibited, while M2 macrophages were significantly activated in the high-risk score subgroup. Furthermore, differences in keratinocytes, NK cells, and osteoblasts were noted between the subgroups. These findings align with previous studies showing that CD4 T cells and M1 macrophages play a role in inhibiting tumor progression, as seen in glioblastoma^35^, and prostate cancer^36^. Noonepalle, et al. demonstrated that macrophage M1 responses induced acute pro-inflammatory reactions immediately following radiotherapy. However, these macrophages transitioned into anti-inflammatory, pro-tumor M2 macrophages in the following days, contributing to cancer resistance^37^. Additionally, Yang et al. reported that lactate facilitates the formation of an immunosuppressive tumor microenvironment by lowering the pH, which accelerates tumor progression and impairs anti-tumor immunity^10^. These findings suggest that lactate-related genes promote radiotherapy resistance by enhancing the Warburg effect and suppressing immune regulation.

Fatty acid desaturase 2 (FADS2), a key gene in the risk score model, was found to be downregulated, indicating a poor prognosis for radiotherapy patients with lung cancer. FADS2 is essential for the biosynthetic pathways that produce long-chain polyunsaturated fatty acids (PUFAs)^25^. PUFAs play important roles in tumor progression, enhancing the efficacy of chemotherapy and radiotherapy while reducing the risk of recurrence by modulating inflammation and immune responses in lung and colorectal cancers^26^, ^38^. Besides these, PUFAs significantly impact mitochondrial oxidative phosphorylation (OXPHOS), stimulating the tricarboxylic acid cycle in the mitochondria to produce more ATP and ROS than glycolysis^39^. Elevated FADS2 expression was shown to inhibit radiotherapy resistance in lung cancer cells, similar to its role in reducing DNA damage in esophageal adenocarcinoma^40^. It is hypothesized that decreased FADS2 expression reduces PUFA production, leading to downregulation of OXPHOS and promoting the Warburg effect, thereby facilitating radiotherapy resistance in lung cancer.

The study has several limitations. Our analysis was based solely on cohorts from TCGA and GEO, and future research should include additional real-world cohorts to further validate the lactate-related risk score. Additionally, further experiments are required to fully elucidate the underlying mechanisms. In summary, a novel lactate-related risk score model for predicting radiotherapy outcomes has been developed, with FADS2 identified as a potent biomarker for predicting radiotherapy resistance in lung cancer. While previous studies have primarily focused on lactate-related genes for prognostic significance in lung cancer, this study is the first to demonstrate their potential utility in predicting radiotherapy efficacy through integrative multi-database analysis. Assessing FADS2 expression in surgical or biopsy specimens imposes no additional burden on patients, underscoring its strong clinical applicability. This study offers a promising therapeutic strategy for clinicians and is likely to stimulate interest among drug developers.

## Supplementary Material

Supplementary tables.

## Figures and Tables

**Table 1:** Univariate analysis of LRDs associated with OS of patients with lung cancer underlying radiotherapy in TCGA.

Characteristics	Univariate analysis	
	HR (95% CI)	P value
FADS2	0.662 (0.537 - 0.816)	< 0.001
PARP1	0.555 (0.369 - 0.834)	0.005
STC2	0.774 (0.648 - 0.924)	0.005
DARS2	0.592 (0.407 - 0.860)	0.006
LMNB1	0.675 (0.507 - 0.897)	0.007
ATP2A2	0.609 (0.424 - 0.873)	0.007
RCC2	0.598 (0.406 - 0.880)	0.009
PHGDH	0.821 (0.708 - 0.952)	0.009
UPB1	1.246 (1.055 - 1.473)	0.010
ASPM	0.773 (0.634 - 0.943)	0.011
PSTPIP1	1.382 (1.066 - 1.792)	0.015
KIF21A	0.703 (0.528 - 0.935)	0.016
CAPN3	1.267 (1.040 - 1.543)	0.019
ALDH18A1	0.664 (0.472 - 0.934)	0.019
HNRNPU	0.496 (0.275 - 0.895)	0.020
MKI67	0.787 (0.637 - 0.972)	0.026
VDAC1	0.625 (0.405 - 0.966)	0.034
RTBDN	0.896 (0.809 - 0.993)	0.036
ADAMTS3	0.848 (0.726 - 0.991)	0.038
PITX3	0.881 (0.780 - 0.995)	0.041
ASIC2	0.852 (0.728 - 0.997)	0.046
ZMPSTE24	0.619 (0.385 - 0.993)	0.047
HSPA4	0.637 (0.406 - 0.998)	0.049

## Abbreviations

DEG: differentially expressed genes
LRDs: lactate-related differentially expressed genes
TCGA: The Cancer Genome Atlas
GO: Gene Ontology
KEGG: Kyoto Encyclopedia of Genes and Genomes
GSEA: Gene set enrichment analysis
GSVA: gene set variation analysis
LASSO: least absolute shrinkage and selection operator
ROC: receiver operating characteristic
IHC: immunohistochemical
qPCR: quantitative real-time PCR
WGCNA: weighted gene co-expression network analysis
TOM: topological overlap matrix
FADS2: Fatty acid desaturases 2
PUFA: polyunsaturated fatty acids
OXPHOS: oxidative phosphorylation
