# Supplementary material for: Investigating the Role of Lactate-Related Genes in Radiotherapy Resistance of Lung Cancer by Integrated Bioinformatics and Experiment Validation
Source: J Cancer. 2025 Jul 24;16(11):3296–313. doi: 10.7150/jca.113046 (PMC12374832; doi:10.7150/jca.113046)
Supplement: Supplementary file 1 — Supplementary tables. [file jcav16p3296s1.pdf]

Supplementary table 1. Primer sequences for the genes.

Gene	Primer	Sequence（5’--3’）
FADS2	Forward	AATCATCGCCACTTCCAGCA
	Reverse	CCCAGAACAAACACGTGCAG
GAPDH	Forward	CCACTAGGCGCTCACTGTTC
	Reverse	CTTCCCGTTCTCAGCCTTGA

Supplementary table 2. Characteristics of lung cancer patients and lactate-related risk score.

Characteristics	All patients	lactate-related risk score (Low)	lactate-related risk score (High)	P value
	n=99	n=50	n=49	
	No.	No.	No.	
**Age at diagnosis (years)**				0.192
<65	48	21	27	
≥65	51	29	22	
**Sex**				＜0.001
female	56	35	21	
male	43	15	28	
**Radiotherapy**				＜0.001
sensitive	35	23	12	
resistant	64	27	37	
**T**				＜0.001
T1	28	15	13	
T2-T4	71	35	36	
**N**				＜0.001
N0	51	25	26	
N1-N3	47	24	23	
NX	1	1	0	
